# Glucose-Induced Glucagon-Like Peptide 1 Secretion Is Deficient in Patients with Non-Alcoholic Fatty Liver Disease

**DOI:** 10.1371/journal.pone.0087488

**Published:** 2014-01-29

**Authors:** Christine Bernsmeier, Anne C. Meyer-Gerspach, Lea S. Blaser, Lia Jeker, Robert E. Steinert, Markus H. Heim, Christoph Beglinger

**Affiliations:** 1 Division of Gastroenterology and Hepatology, University Hospital Basel, Basel, Switzerland; 2 Department of Biomedicine, University Hospital Basel, Basel, Switzerland; Sezione di Gastroenterologia, Italy

## Abstract

**Background & Aims:**

The incretins glucagon-like peptide-1 (GLP-1) and glucose-dependent insulinotropic polypeptide (GIP) are gastrointestinal peptide hormones regulating postprandial insulin release from pancreatic β-cells. GLP-1 agonism is a treatment strategy in Type 2 diabetes and is evaluated in Non-alcoholic fatty liver disease (NAFLD). However, the role of incretins in its pathophysiology is insufficiently understood. Studies in mice suggest improvement of hepatic steatosis by GLP-1 agonism. We determined the secretion of incretins after oral glucose administration in non-diabetic NAFLD patients.

**Methods:**

N = 52 patients (n = 16 NAFLD and n = 36 Non-alcoholic steatohepatitis (NASH) patients) and n = 50 matched healthy controls were included. Standardized oral glucose tolerance test was performed. Glucose, insulin, glucagon, GLP-1 and GIP plasma levels were measured sequentially for 120 minutes after glucose administration.

**Results:**

Glucose induced GLP-1 secretion was significantly decreased in patients compared to controls (p<0.001). In contrast, GIP secretion was unchanged. There was no difference in GLP-1 and GIP secretion between NAFLD and NASH subgroups. All patients were insulin resistant, however HOMA2-IR was highest in the NASH subgroup. Fasting and glucose-induced insulin secretion was higher in NAFLD and NASH compared to controls, while the glucose lowering effect was diminished. Concomitantly, fasting glucagon secretion was significantly elevated in NAFLD and NASH.

**Conclusions:**

Glucose-induced GLP-1 secretion is deficient in patients with NAFLD and NASH. GIP secretion is contrarily preserved. Insulin resistance, with hyperinsulinemia and hyperglucagonemia, is present in all patients, and is more severe in NASH compared to NAFLD. These pathophysiologic findings endorse the current evaluation of GLP-1 agonism for the treatment of NAFLD.

## Introduction

Non-alcoholic fatty liver disease (NAFLD) has become the most frequent chronic liver disease in Western countries. Non-alcoholic steatohepatitis (NASH) is regarded as a subgroup of NAFLD defined by histological coexistence of hepatic steatosis and inflammation. However, some authors postulate different conditions of diseases for NAFLD and NASH [1], [2]. NASH, but not NAFLD, carries the risk of disease progression and complications such as cirrhosis, liver failure or hepatocellular carcinoma (HCC). NAFLD and NASH are associated with obesity, insulin resistance (IR) and type 2 diabetes mellitus (T2DM). The recommended treatment consists of diet combined with exercise, aiming at weight loss and improvement of insulin sensitivity. To date, no standardized pharmacological treatment has been approved [2], [3].

The main events in the pathophysiology of NAFLD/NASH are hepatic lipid accumulation, lipotoxicity and inflammation [4]. Hepatic lipid accumulation is due to different mechanisms, mainly increased dietary intake, de-novo lipogenesis and influx of free fatty acids (FFA). A major risk factor is IR. IR induces lipolysis in adipose tissues, hereby increasing flux of FFA into the liver [5].

The incretins, glucagon-like peptide-1 (GLP-1) and glucose-dependent insulinotropic polypeptide (GIP) are gastrointestinal peptide hormones regulating postprandial insulin release from pancreatic β-cells, the so-called “incretin effect”[6]. GLP-1 is released postprandially from endocrine L-cells into the splanchnic and portal circulation. It lowers plasma glucose and improves insulin sensitivity by increasing postprandial insulin release, decreasing glucagon secretion and delaying gastric emptying [7]. Additional effects include reduction in energy intake, increase in satiety [8], and weight loss [9]. GLP-1 agonism is an approved treatment strategy in T2DM using GLP-1 receptor (GLP-1R) agonists and dipeptidyl peptidase-4 (DPP-4) inhibitors [10].

The role of incretins in NAFLD is insufficiently understood. Experimental data suggest a link between GLP-1 and steatogenesis. Prevention or reversal of hepatic steatosis by different GLP-1 agonists including exendin-4 [11], [12], liraglutide [13], [14], GLP-1(28–36)amide [15], and AC3174 [16] has been shown in rodents. There is evidence for the presence of GLP-1R on human hepatocytes [12], [17]. The mechanism of GLP-1 mediated inhibition of hepatic lipid accumulation is unknown. It probably involves diverse signalling pathways regulating lipogenesis [14], [17], [18], ER stress and autophagy [19].

Despite the evidence in murine models, data in humans is limited. Down-regulation of GLP-1R [17] and up-regulation of DPP-4 [20] have been reported in liver biopsies of NAFLD patients. Recently, improvement of lipid accumulation, assessed by liver proton magnetic resonance spectroscopy (^1^H-MRS), has been shown in patients with T2DM treated with GLP-1 agonists [21].

In NAFLD patients, neither secretion of incretins nor GLP-1R signalling has been studied to date. We hypothesized that deficiency in GLP-1 secretion might maintain hepatic steatosis. Thus, the aim of this study was to determine incretin secretion in patients with NAFLD and NASH. In our cohort of 52 non-diabetic patients with NAFLD and NASH, we newly demonstrate deficiency of glucose-induced GLP-1 secretion. We hereby add to the limited knowledge on the role of incretins in the pathophysiology of NAFLD/NASH in humans.

## Methods

### Ethics Statement

The protocol conforms to ethical guidelines of the 1975 Declaration of Helsinki and has been approved by the ethics committee of the Kanton Basel (Ethikkommission beider Basel). Written informed consent was obtained from all participants. The study was registered at www.clinicaltrials.gov (NCT01674972).

### Patients

The study included 52 non-diabetic patients with biopsy proven NAFLD, including patients with simple steatosis (n = 16) or NASH (n = 36) and 50 healthy controls. Patients were subjects of the Basel NAFLD cohort. Liver biopsies had previously been obtained and evaluated by a hepato-pathologist. Differentiation between NAFLD and NASH was done using the NAFLD activity score [22]. Exclusion criteria for NAFLD patients were alcohol consumption >40g/d for male and >20g/d for female subjects, concomitant liver disease and T2DM.

Healthy controls were matched by sex and screened for the metabolic syndrome and liver disease by medical history and a blood sample including liver function tests (LFT), fasting glucose and lipids. Exclusion criteria for controls were alcohol consumption (criteria stated above), smoking, any liver disease, elevated LFTs, elevated lipids, IR or intake of any drug with a known influence on glucose homeostasis.

### Insulin resistance

IR was assessed calculating the homeostasis model assessment HOMA2-IR [23].

### Oral glucose tolerance test

Oral glucose tolerance test (oGTT) was performed in all subjects after an overnight fast according to a standardized protocol using 75 g of glucose in 300 ml tap water (300 kcal). Baseline vital parameters, height, weight and BMI were taken. Blood samples were drawn before and sequentially at 15, 30, 60, 90 and 120 minutes after glucose administration. Collection tubes contained EDTA (6 µmol/l), aprotinin (500 kIU/l) and a DPP-4 inhibitor, and were kept on ice. Plasma samples were stored at −70°C for subsequent assessment of GLP-1, GIP, fasting glucose, insulin, glucagon and baseline LFTs.

### Hormones, glucose and liver function tests

GLP-1 was measured using an ELISA kit (Linco Research, St. Charles, USA) as previously reported [24]. This kit is highly specific for biologically active forms of GLP-1 (i.e., 7–36 amide and 7–37) and will not detect other forms (e.g., 1–36 amide, 1–37, 9–36, or 9–37). Values are expressed as pmol/l. The sensitivity of the assay is 2 pmol/l. GIP was measured using an ELISA kit (EMD Millipore, Billerica, USA). It detects human GIP(1–42) and GIP(3–42). Values are expressed as pg/ml. The sensitivity is 4.2 pg/ml.

Glucagon (Siemens, Malvern, USA), sensitivity: 13 pg/ml, and insulin (Cisbio International, Bagnols, France), sensitivity: 4.6 µU/ml, were measured by radioimmunoassay. Blood glucose concentrations were measured using hexokinase-method (Roche, Basel, Switzerland). LFTs were assessed by routine diagnostics by standardized IFCC protocol using pyridoxale-5phosphate.

### Statistical analysis

Statistical analysis was done using SPSS software (V.19.0 for Windows, SPSS Inc., Chicago, USA). Data are presented as mean±SEM. For data that did not follow a normal distribution, the significance of differences was tested using Mann-Whitney U and multiple Mann-Whitney tests with Bonferroni-Holm adjustment of p-values for multiplicity of testing. Differences were considered to be significant at p<0.05. Graphs were drawn using GraphPad Prism 6.0c for Macintosh, GraphPad Software, San Diego, USA.

## Results

### Baseline characteristics

NAFLD patients and healthy controls were matched by sex (male sex was predominant in both groups, 69.2% and 68.0% respectively). NAFLD patients were divided into a NAFLD-subgroup, including patients with simple steatosis, and a NASH-subgroup. Age was higher in the patient groups compared to controls.

Weight and BMI were significantly higher in both NAFLD and NASH patients compared to controls and demonstrate pre-obesity, according to the WHO definitions.

LFTs were pathologic in both NAFLD and NASH, whilst values were higher in the NASH subgroup. The difference between NAFLD and NASH subgroups was significant for aspartate aminotransferase (ASAT) only (p = 0.0044) (Table 1).

**Table 1:** Baseline characteristics of Non-alcoholic fatty liver disease (NAFLD) and Non-alcoholic steatohepatitis (NASH) patients and controls.

	Patients			Controls	Patients vs. Controls	NASH vs. NAFLD
	all	NAFLD	NASH			
Age (years)	48.9±2.0	48.6±2.7	49.1±2.7	35.9±1.9	*p<0.0001*	*ns*
Weight (kg)	85.7±2.1	82.9±2.9	87.0±2.8	71.0±1.1	*p<0.0001*	*ns*
BMI (kg/m^2^)	29.1±0.6	28.5±1.0	29.3±0.8	22.7±0.2	*p<0.0001*	*ns*
Fasting glucose (mmol/l)	5.0±0.1	5.0±0.1	5.0±0.1	5.2±0.1	*p = 0.0071*	*ns*
Fasting insulin (mU/l)	38.9±3.1	27.8±2.3	43.9±4.1	14.9±0.9	*p<0.0001*	*p = 0.0033*
HOMA2-IR	4.7±0.3	3.5±0.3	5.2±0.4	1.9±0.1	*p<0.0001*	*p = 0.0044*
Fasting glucagon (pg/ml)	52.0±6.1	42.9±10.5	56.1±7.5	25.8±1.7	*p<0.001*	*ns*
ASAT (U/l)	44.6±4.4	30.6±2.6	50.9±6.0	26.8±1.1	*p = 0.0001*	*p = 0.0044*
ALAT (U/l)	61.5±6.6	46.3±5.9	68.3±9.0	21.8±1.3	*p<0.0001*	*ns*
GGT (U/l)	82.5 ±10.3	66.7±9.2	89.6±14.3	22.8±2.8	*p<0.0001*	*ns*

Data are expressed as mean±SEM. n = 52 patients (NAFLD n = 16 (30.8%), NASH n = 36 (69.2%)); n = 50 controls. Differences in baseline characteristics of patients vs. controls and NAFLD vs. NASH are expressed as p-values (Mann-Whitney U test). P≤0.05, statistically significant difference; ns, not significant.

### Insulin resistance

Fasting glucose levels were within normal range in all subjects and were not significantly higher in our non-diabetic patients. Fasting insulin levels in patients with NAFLD and NASH were, however, significantly higher compared to controls (27.8±2.3 and 43.9±4.1 [all patients 38.9±3.1] vs. 14.9±0.9 mU/l, p<0.0001). Assessment of IR by HOMA2-IR suggests IR in patients but not controls (4.7±0.3 vs. 1.9±0.1, p<0.0001). IR was significantly higher in the NASH compared to the NAFLD subgroup (p = 0.0044) (Table 1, Table S1).

Also fasting glucagon levels were significantly elevated in patients compared to controls (52.0±6.1 vs. 25.8±1.7, p<0.001). There was no significant difference between the NAFLD and NASH subgroups.

### Glucose-induced secretion of incretins GLP-1 and GIP

**Figure 1:**
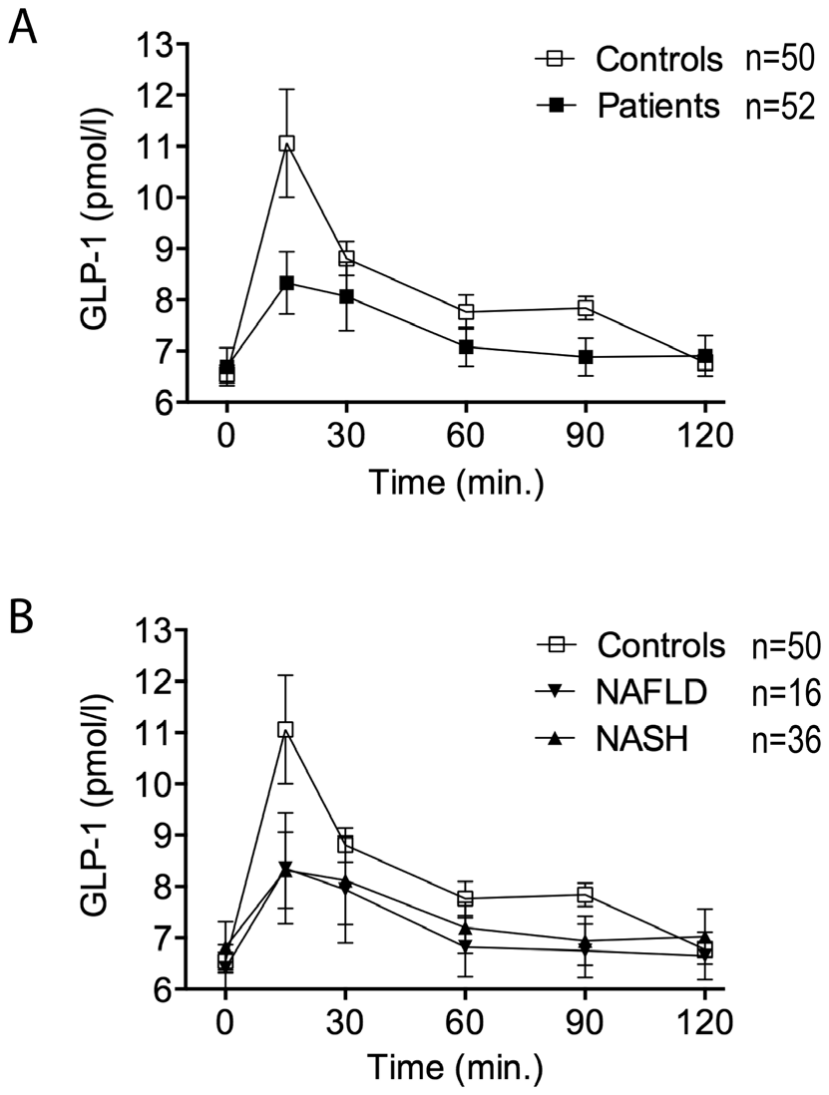
Glucagon-like peptide 1 (GLP-1) secretion in response to oral glucose tolerance test (oGTT). **(A)** GLP-1 secretion in response to oGTT is significantly decreased in patients vs. controls (p<0.001). Patients n = 52; controls n = 50. **(B)** GLP-1 secretion in NAFLD and NASH vs. controls. NAFLD n = 16; NASH n = 36; controls n = 50. GLP-1 (pmol/ml) is expressed as mean±SEM.

Mean fasting GLP-1 plasma levels did not differ between the groups. After oral glucose administration, GLP-1 concentrations peaked at 15 minutes in all groups. Peak GLP-1 concentrations were significantly lower in patients compared to controls (p<0.001), as was total GLP-1 secretion over 120 minutes (p<0.001) (Table 2; Figure 1A). There was no difference in glucose induced GLP-1 secretion between the NAFLD and NASH subgroups (Table 3; Figure 1B). Also there was no significant difference in GLP-1 secretion when comparing patients with BMI>30 kg/m^2^ (n = 18) to those with BMI<30 kg/m^2^ (n = 34) (p = 0.4039).

**Table 2:** Secretion of Glucagon-like peptide-1 (GLP-1), Glucose-dependent insulinotropic polypeptide (GIP), insulin and glucagon and glucose disposal in patients with NAFLD and NASH vs. controls in response to oral glucose tolerance test.

	Patients	Controls	
**GLP-1**			
AUC (0–120 min) (pmol × min/l)	879±53	969±34	*p<0.001*
c_max_ (pmol/l)	8.8±0.7	11.7±1.0	*p<0.001*
**GIP**			
AUC (0–120 min) (pg × min/ml)	23348±1329	22880±1397	*ns*
c_max_ (pg/ml)	263.2±15.3	248.6±15.9	*ns*
**Glucose**			
AUC (0–120 min) (mmol × min/l)	962 ±28	795±24	*p<0.001*
c_max_ (mmol/l)	9.7±0.3	8.6 ±0.3	*p = 0.007*
Δ **Insulin**			
AUC (0–120 min) (mU × min/L)	10579±456	5981±308	*p<0.001*
c_max_ (mU/L)	118.9±3.9	91.5±5.1	*p<0.001*
**Glucagon**			
AUC (0–120 min) (pg × min/ml)	4489±526	2947±150	*p = 0.014*
c_max_ (pg/ml)	62.3±6.5	32.2±1.6	*p<0.001*

Patients n = 52; controls n = 50. Data are expressed as mean±SEM. AUC, area under the curve; c_max_, maximum plasma concentration. Multiple Mann-Whitney tests with Bonferroni-Holm adjustment of p-values for multiplicity of testing. P≤0.05, statistically significant difference; ns, not significant.

**Table 3:** Secretion of Glucagon-like peptide-1 (GLP-1), Glucose-dependent insulinotropic polypeptide (GIP), insulin and glucagon and glucose disposal in NAFLD and NASH subgroups vs. controls in response to oral glucose tolerance test.

	NAFLD	NASH	NASH vs. Controls	NAFLD vs. Controls	NASH vs. NAFLD
**GLP-1**					
AUC (0–120 min) (pmol × min/l)	859±78	888±68	*p = 0.003*	*ns*	*ns*
c_max_ (pmol/l)	8.8±1.1	8.7±0.9	*p = 0.001*	*ns*	*ns*
**GIP**					
AUC (0–120 min) (pg × min/ml)	22328±1663	23802±1781	*ns*	*ns*	*ns*
c_max_ (pg/ml)	262.3±24.3	263.7±19.5	*ns*	*ns*	*ns*
**Glucose**					
AUC (0–120 min) (mmol × min/l)	894±51	993±32	*p<0.001*	*ns*	*ns*
c_max_ (mmol/l)	9.1±0.6	10.0±0.3	*p = 0.009*	*ns*	*ns*
Δ **Insulin**					
AUC (0–120 min) (mU × min/l)	9209±902	11188±499	*p<0.001*	*p = 0.003*	*p = 0.054 (ns)*
c_max_ (mU/l)	116.8±8.3	119.9±4.3	*p<0.001*	*p = 0.051 (ns)*	*ns*
**Glucagon**					
AUC (0–120 min) (pg × min/ml)	3986±819	4713±670	*p = 0.011*	*ns*	*ns*
c_max_ (pg/ml)	56.5±10.9	64.9±8.2	*p<0.001*	*p = 0.031*	*ns*

NAFLD n = 16; NASH n = 36; controls n = 50. Data are expressed as mean±SEM. AUC, area under the curve; c_max_, maximum plasma concentration. Multiple Mann-Whitney tests with Bonferroni-Holm adjustment of p-values for multiplicity of testing. P≤0.05, statistically significant difference; ns, not significant.

**Figure 2:**
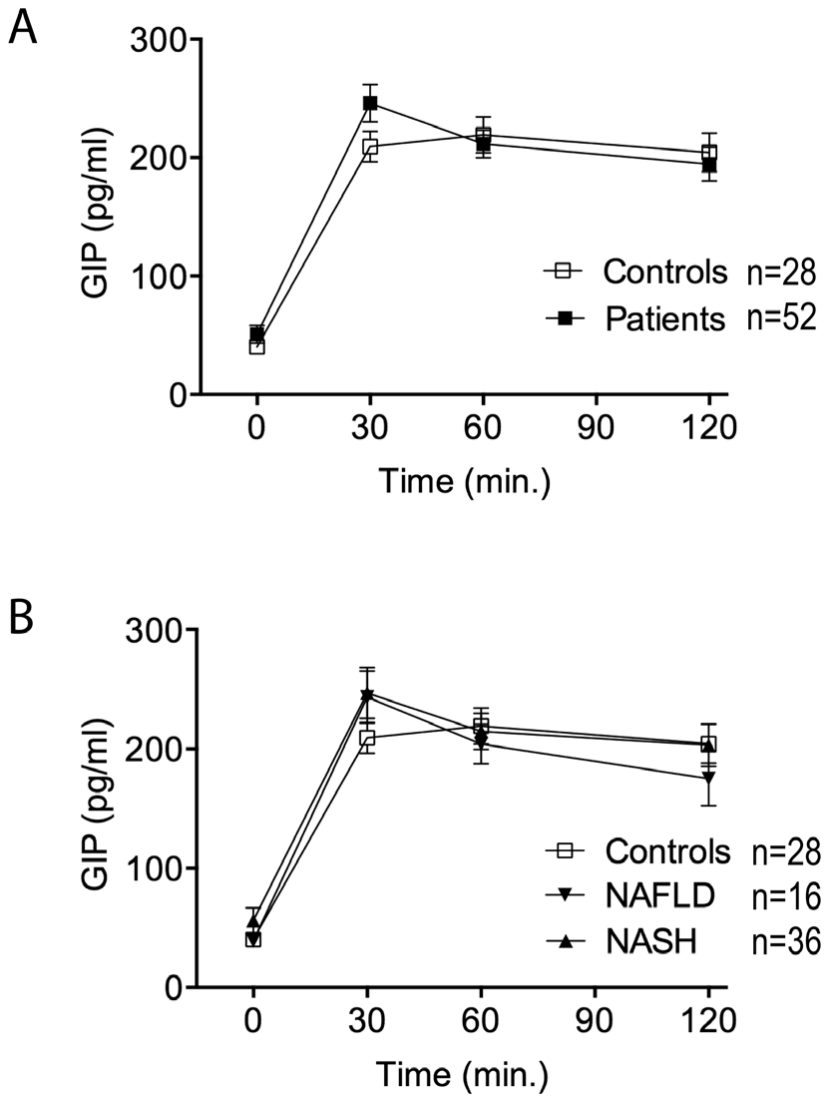
Glucose-dependent insulinotropic polypeptide (GIP) secretion in response to oGTT. **(A)** GIP secretion in response to oGTT is not different in patients vs. controls. Patients n = 52; controls n = 28. **(B)** GIP secretion in NAFLD and NASH vs. controls. NAFLD n = 16; NASH n = 36; controls n = 28. GIP (pg/ml) is expressed as mean±SEM.

Mean fasting GIP plasma levels were not different between the groups. Moreover, after oGTT neither peak nor total GIP concentrations differed significantly between patients and controls (Table 2; Figure 2A) or NAFLD and NASH subgroups (Table 3; Figure 2B).

### Glucose-induced glucose disposal, insulin and glucagon secretion

**Figure 3:**
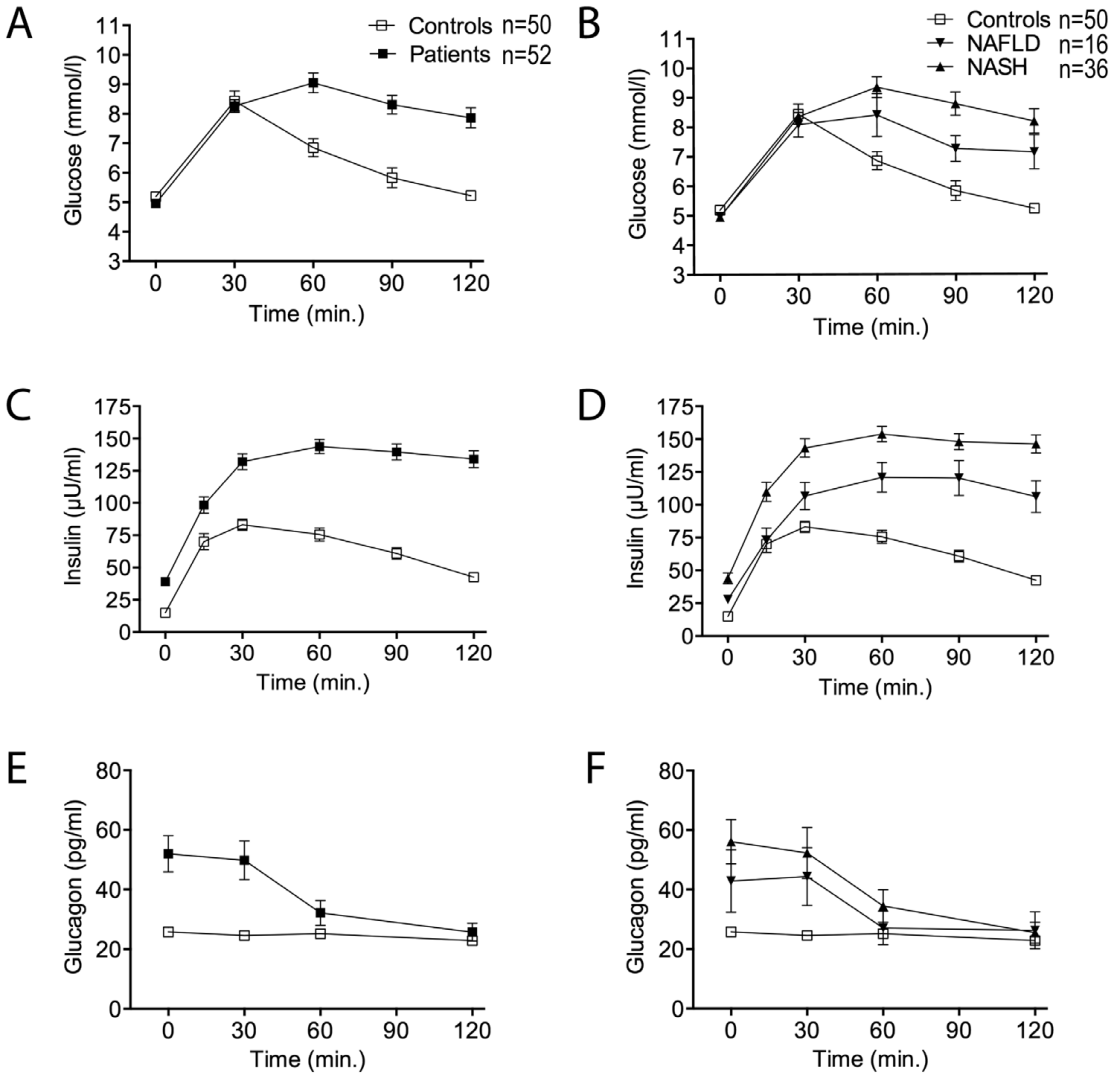
Glucose, insulin and glucagon concentrations in response to oGTT. Plasma glucose **(A, B)**, insulin **(C, D)** and glucagon **(E, F)** concentration curves are shown for patients vs. controls **(A, C, E)** and NAFLD and NASH vs. controls **(B, D, F)**. Patients n = 52 (NAFLD n = 16; NASH n = 36); controls n = 50. Glucose (mmol/l), insulin (µU/ml) and glucagon (pg/ml) are expressed as mean±SEM.

Figure 3 shows time courses for plasma glucose, insulin and glucagon in response to oral glucose administration.

Glucose disposal after oGTT was significantly reduced and delayed in patients compared to controls (Table 2; Figure 3A). Comparing the NASH and NAFLD subgroups, glucose disposal was numerically but not significantly lower in NASH (Table 3; Figure 3B).

Moreover patients showed a pathologic insulin release after oGTT. Insulin levels increased to significantly higher levels and decreased slower in patients compared to controls (ΔInsulin) (Table 2; Figure 3C). Insulin release was significantly higher in both subgroups compared to controls (p<0.001 and p = 0.003, respectively) (Table 3). NASH patients showed higher insulin release than NAFLD patients (Table 3; Figure 3D). The numeric difference between the subgroups does, however, not reach statistical significance (p = 0.054) (Table 3).

Elevated baseline glucagon levels in patients decreased slowly during oGTT, reaching levels comparable to the control group after 120 minutes. Thus, maximal and total glucagon levels were significantly higher in patients compared to controls (Table 2; Figure 3E). Decrease in plasma glucagon levels tended to be faster in the NAFLD compared to the NASH subgroup (Figure 3F), however the difference was not statistically significant (Table 3; Figure 3F).

## Discussion

The growing prevalence of NAFLD/NASH and the growing impact of NASH related complications on the treatment of end-stage liver disease, HCC and health economy in general ask for elucidation of the pathophysiologic mechanisms involved in order to develop pharmacological treatment strategies.

The results of this study newly document deficiency of glucose-induced GLP-1 secretion in patients with NAFLD and NASH. The data adds to the limited knowledge of incretins in the pathophysiology of human NAFLD and substantiate the hypothesis that the GLP-1/GLP-1R system is impaired. This hypothesis had been raised by previous studies in rodents linking GLP-1 agonism to reversal of steatosis [11], [13]–[19], but validation in humans is missing. Our study might further endorse the investigation of GLP-1 analogues as a novel therapeutic approach.

Studying the pathophysiology of steatosis and steatohepatitis in rodents is difficult and there is no model that sufficiently reflects human pathogenesis of NAFLD/NASH. Models using genetic modifications such as ob/ob and db/db mice as well as methionine-choline deficient diet are not comparable to the pathophysiology of NAFLD. In mice with steatosis induced by a diet rich in trans-fat and fructose, GLP-1 analogues previously reversed lipid accumulation [13], [16]. Trevaskis et al. further demonstrated that reversibility of steatosis was dependent on hepatic GLP-1R, that had been found down-regulated in NAFLD patients [16]. These observations merit validation in human studies.

Whether or not GLP-1 secretion is deficient in patients with T2DM has been debated: earlier studies had found low GLP-1 secretion in poorly controlled diabetics [25], [26] while others did not [27]. The current knowledge suggests that the impaired incretin effect in T2DM is due to attenuated postprandial GLP-1 response, decreased insulinotropic effect of GLP-1 and an almost complete loss of insulin secretion in response to GIP [7], [28]. From clinical studies, there is substantial evidence that incretin-based therapies exhibit various beneficial effects in patients with T2DM including correction of dyslipidaemia and prevention of weight gain apart from their glucose-lowering properties [29].

NAFLD is strongly associated with IR and obesity. Again, it is debated whether GLP-1 secretion is impaired in insulin-resistant subjects. Some studies found impaired early GLP-1 secretion in response to a mixed meal or glucose in IR [30], [31], others reported normal GLP-1 secretion [27]. There is however, some evidence that GLP-1 secretion is not impaired in obese compared to lean subjects [32]–[34].

In our cohort of non-diabetic NAFLD/NASH patients, we observed impaired GLP-1 secretion in response to oGTT with consequences for insulin, glucagon and glucose dynamics in comparison to a healthy control group. The magnitude of reduction in glucose-induced GLP-1 secretion is striking.

Due to the nature of NAFLD/NASH, diseases related to obesity and IR, we were not able to match the control group for these factors. To address the possibility that impaired GLP-1 secretion might be explained by obesity alone, we did subgroup analysis according to BMI. GLP-1 secretion in obese compared to pre-obese subjects was not statistically different, demonstrating that BMI might influence but not explain the discrepancy in GLP-1 secretion.

A limitation of this study is the higher age of patients compared to controls. It would have been ideal to compare to a healthy, non-obese, non-insulin resistant cohort with a mean age of 49 years but would have implied high screening and dropout numbers in our western population. We did not insist on this criterion as previous studies had shown that glucose induced GLP-1 secretion is independent of age [35], [36].

The observed deficiency in glucose induced GLP-1 secretion in NAFLD/NASH seems therefore to be related to hepatic steatosis.

Liraglutide and exenatide have been shown to reduce hepatic lipid accumulation in diabetics, measured non-invasively by ^1^H-MRS [21]. The therapeutic effect of GLP-1 agonists on non-diabetic patients with NAFLD/NASH is currently studied in clinical trials (www.clinicaltrials.gov) [37]. It is heretofore unknown whether GLP-1 deficiency might explain the above described therapeutic effect. In addition to the deficiency in GLP-1 secretion observed here, the insulinotropic effect might be deficient in NAFLD. Evidence from studies in mice [11], [13]–[19] suggest the presence of “hepatic GLP-1-resistance” at the GLP-1R level or downstream. Consistent with this assumption, reduced GLP-1R expression [17] and DPP-4 up-regulation [20] have been reported in liver tissue of NAFLD patients. It therefore remains unclear whether deficiency in GLP-1 secretion, hepatic GLP-1 resistance or both components are involved.

In this study IR is present in NAFLD/NASH patients and mean HOMA2-IR is higher in NASH compared to NAFLD. The data confirms results of previous studies [38].

Whereas the role of insulin and IR have been extensively studied, the important role of glucagon in regulating glucose homeostasis has been neglected and re-discovered only recently [39], [40]. Inappropriate hyperglucagonemia is observed in diabetes and IR. Our data clearly show a synergism of hyperinsulinemia and hyperglucagonemia in the context of NAFLD/NASH. Inhibition of glucagon secretion by oral glucose is markedly delayed.

Interestingly, and unlike IR, GLP-1 secretion is impaired to the same extent in both NAFLD and NASH subgroups. This allows two possible conclusions: first, impaired GLP-1 secretion is probably associated with the cause or consequence of steatogenesis, as suggested by previous experimental studies [11]–[13], [15]–[19] and is seen as an early event in the pathogenesis of NAFLD. In accordance with the literature our results do not link GLP-1 deficiency to hepatic inflammation. Second, impaired GLP-1 secretion is not related to severity of IR. However we cannot clarify the contentious issue whether or not GLP-1 secretion is impaired in IR alone. To address this question, a study comparing patients with IR alone and patients with IR and NAFLD would be instructive.

Deficiency of GLP-1 secretion is supposed to decrease glucose-induced insulin secretion and consequently delay the glucose-lowering effect. Controversially, in our cohort, impaired GLP-1 secretion parallels delayed glucose-lowering effect but increased and prolonged insulin secretion. This observation implicates that GLP-1 is not the unique regulator of glucose-induced insulin secretion in NAFLD. In accordance with previous data for subjects with IR and T2DM [27], [28], we demonstrate that GIP secretion is unchanged in NAFLD patients. Thus, GIP is unlikely to explain glucose-induced hyperinsulinemia. Possible explanations are involvement of other incretins or non-hormonal regulators of insulin secretion, or the compensatory hyperplasia of β-cells and hypersecretion of insulin in the insulin-resistant state [41], [42]. The influence of these factors respectively needs to be investigated further.

In conclusion, we demonstrate deficiency of glucose-induced GLP-1 secretion in a cohort of NAFLD/NASH patients. Impairment of GLP-1 secretion in both NAFLD and NASH supports a role for GLP-1 in steatogenesis that had been proposed from rodent models. The discordance between GLP-1 secretion and insulin release implicate a more complex model involving interplay with other regulators of insulin secretion, e.g. non-hormonal mediators, or modulation of β-cell sensitivity that needs to be further explored. Impaired GLP-1 secretion is a component of impaired GLP-1/GLP-1R system in patients with NAFLD, additional to a proposed GLP-1 resistance at the tissue level. This knowledge might emphasize the possible therapeutic value of GLP-1 agonism in patients with NAFLD. However, additional experimental studies are needed to elucidate the mechanisms of impaired GLP-1/GLP-1R system in the pathophysiology of NAFLD.

## Supporting Information

Table S1 Differences in baseline characteristics of NAFLD and NASH vs. controls. NAFLD n = 16 (30.8%), NASH n = 36 (69.2%); n = 50 controls. Data are expressed as p-values (Mann-Whitney U test). P≤0.05, statistically significant difference; ns, not significant.
(DOCX)
