# Supplementary material for: Glucose-Induced Glucagon-Like Peptide 1 Secretion Is Deficient in Patients with Non-Alcoholic Fatty Liver Disease
Source: PLoS One. 2014 Jan 29;9(1):e87488. doi: 10.1371/journal.pone.0087488 (PMC3906180; doi:10.1371/journal.pone.0087488)
Supplement: Table S1 — Differences in baseline characteristics of NAFLD and NASH vs. controls. NAFLD n = 16 (30.8%), NASH n = 36 (69.2%); n = 50 controls. Data are expressed as p-values (Mann-Whitney U test). P≤0.05, statistically significant difference; ns, not significant. (DOCX) [file pone.0087488.s001.docx]

**Table S1**

|  | **NASH vs. Controls** | **NAFLD vs. Controls** |
| --- | --- | --- |
|  |  |  |
| Weight (kg) | *p<0.0001* | *p=0.0004* |
| BMI (kg/m2) | *p<0.0001* | *p<0.0001* |
|  |  |  |
| Fasting glucose (mmol/l) | *p=0.0133* | *p=0.0755* |
| Fasting insulin (mU/l) | *p<0.0001* | *p<0.0001* |
| HOMA2-IR | *p<0.0001* | *p<0.0001* |
| Fasting glucagon (pg/ml) | <0.0001 | ns |
|  |  |  |
| ASAT (U/l) | *p<0.0001* | *ns* |
| ALAT (U/l) | *p<0.0001* | *p<0.0001* |
| GGT (U/l) | *p<0.0001* | *p<0.0001* |

**Table S1. Differences in baseline characteristics of NAFLD and NASH vs. controls.** NAFLD n=16 (30.8%), NASH n=36 (69.2%); n=50 controls. Data are expressed as p-values (Mann-Whitney U test). P≤0.05, statistically significant difference; ns, not significant.
